# Effect of D-ring C-3’ methylation of strigolactone analogs on their transcription regulating activity in rice

**DOI:** 10.1080/15592324.2019.1668234

**Published:** 2019-09-25

**Authors:** Muhammad Jamil, Imran Haider, Boubacar A. Kountche, Salim Al-Babili

**Affiliations:** Division of Biological and Environmental Sciences and Engineeringthe, BioActives Lab, King Abdullah University of Science and Technology, Thuwal, Saudi Arabia

**Keywords:** Strigolactones, methylation, gene expression, rice

## Abstract

Strigolactones (SLs) are a well-known class of plant hormones, which are involved in a number of developmental and adaptation processes and mediate different interspecific interactions. In spite of the growing knowledge on SL biosynthesis and signal transduction, effects of structural modifications on the activity and efficiency of SLs and their analogs remain largely elusive. SLs are characterized by the presence of a lactone ring (D-ring) that is connected by an enol ether bridge to a second moiety. In this study, we investigated the effect of additional D-ring methylation of SL analogs on their transcription regulating activity. For this purpose, we compared the SL analogs MP13 and AR8, which differ only by the presence of a methyl group at the C-3ʹ atom in the latter. Transcription regulating activity was determined by quantitative real-time PCR measurement of transcript levels of SL-dependent, feed-back regulated genes in treated wild type and *ccd7* mutant rice seedlings. Results obtained indicate that C-3ʹ methylation reduces the transcription regulating activity, as shown by the more pronounced suppression of the SL biosynthesis genes *DWARF27* (*D27*) and *CAROTENOID CLEAVAGE DIOXYGENASES* (*CCD7* and *CCD8*) and higher induction of the SL signaling repressor gene *DWARF53* (*D53*) in MP13 treated seedlings. These results are consistent with a recent study on the biological activities of MP13 and AR8.

Strigolactones (SLs) are a class of carotenoid-derived plant hormones, which controls many aspects of plant architecture and is involved in several developmental processes, including tillering, branching, leaf shape, leaf senescence, internode growth, shoot branching angle, stem secondary growth, and the development of primary, lateral, and adventitious roots and root hairs.^1,2^ In addition, SLs contribute to plant response to biotic and abiotic stresses.^3^ Besides these hormonal functions, SLs act as rhizospheric signaling molecules, mediating the interaction with Arbuscular Mycorrhizal (AM) fungi by inducing hyphal branching and with root parasitic plants by triggering the germination of their seeds.^4,5^

Previous studies have shown that an iron-containing carotenoid isomerase DWARF27 (D27) and two CAROTENOID CLEAVAGE DIOXYGENASES (CCD7 and CCD8) are sequentially required to produce the SL precursor carlactone (CL) from all-*trans*-β-carotene in plastids (Figure 1). Several orthologs of genes encoding CCD7 and CCD8 have been described in a number of different species (MAX3 and MAX4 in *Arabidopsis*, RMS5 and RMS1 in pea and D17 and D10 in rice).^6-9^ Further conversion of CL in the cytosol by a cytochrome P450 enzymes of the 711 clade (MAX1 in *Arabidopsis*) leads to different SLs,^10,11^ which can be modified by further enzymes.^12^ At present, about 25 natural SLs have been identified and characterized from different species, which are classified as either canonical or non-canonical SLs.^13^ Canonical SLs, such as strigol and orobanchol, consist of a typical butenolide lactone D-ring which is linked in *R* configuration to a second moiety, a tricyclic lactone (ABC-ring), while, non-canonical SLs, like carlactonoic acid, comprise less conserved second moiety instead of the ABC-ring.^13,14^ F-box leucine-rich protein (MAX2/RMS4/D3 in *Arabidopsis*, pea and rice, respectively) and α/β-fold hydrolases (D14 in rice) have been found to be involved in SL perception and downstream signaling.^15-17^ After binding of the SL ligand to the receptor, the SCF complex (MAX2 or D3) along with α/β-hydrolase receptor (D14 or DAD2 in pea) triggers the ubiquitination of transcriptional repressors (D53 and SMXLs), resulting in the 26S proteasomal degradation of these repressors, and consequently in stimulating the transcription of SL responsive genes.^18,19^ The biosynthesis of SLs is auto-regulated by a negative feedback mechanism that maintains their homeostasis which is also governed by a complicated network of other plant hormones.^13,20^ In addition, there are a number of factors, particularly nutrient availability, affecting SL biosynthesis. However, it is largely unknown whether structural changes in SLs have an impact on their activity in determining the expression of their own biosynthetic genes and in regulating gene expression in general. In this study, we examined the effect of methylation at the C-3ʹ in the D-ring of SL analogs on their activity in determining the transcript levels of genes involved in SL biosynthesis and perception, using roots of rice seedlings treated with AR8^21^ MP13^22^ two analogs that differ in C-3ʹ methylation, and, as a control, GR24, the common SL analog (Figure 2). Assuming that SL deficient mutant may be more sensitive to SL treatment, we also investigated the effect of these analogs on the *hl-2* (*high-tillering dwarf1-like*), a *ccd7* mutant.^23^

**Figure 1.**
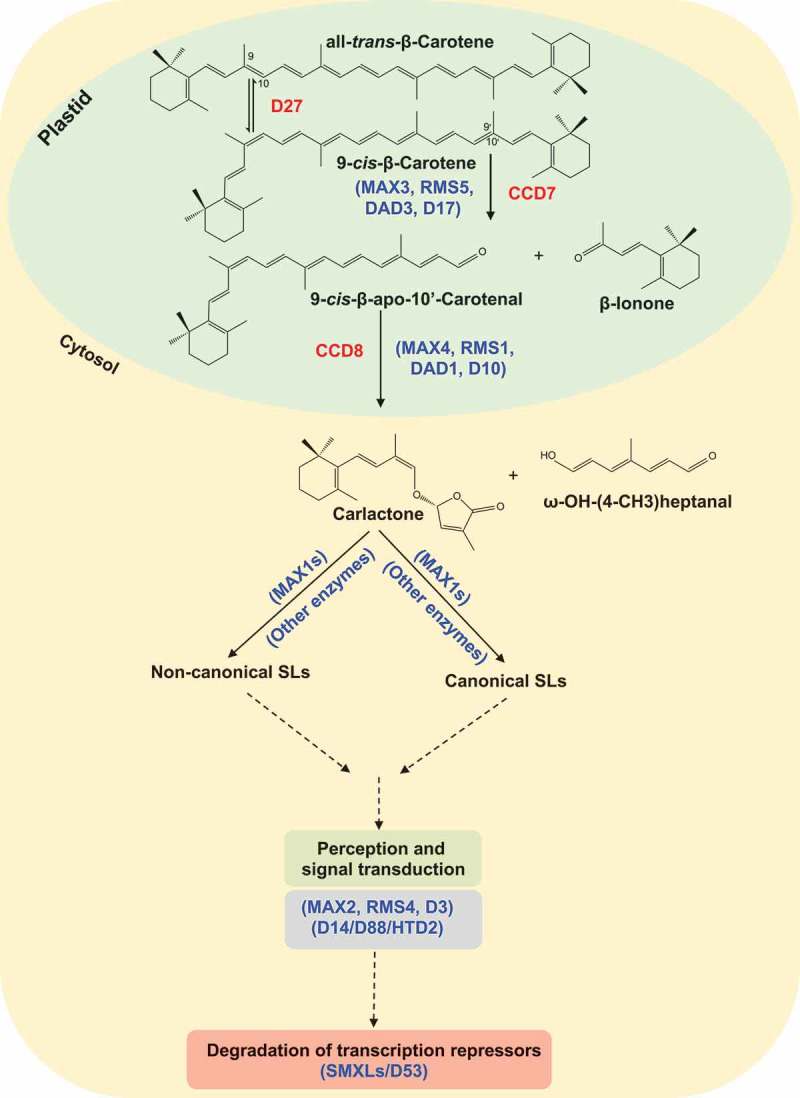
Biosynthesis, perception and signaling pathways of strigolactones (Adapted and modified from^6^). SL biosynthesis involves sequential actions of an isomerase DWARF27 (D27) and two CAROTENOID CLEAVAGE7^7^ (CCD7) and CCD8, which take place within plastids, and Cytochrome P450 (711 clade) and other enzymes in the cytosol (red enzymes on solid arrows). D27 catalyzes the reversible isomerization of all-*trans*- into 9-*cis*-β-carotene that is converted by CCD7 and CCD8 into carlactone, a key intermediate in SL biosynthesis. Cytochrome P450 enzymes (MAX1) in the cytosol further convert carlactone into non-canonical and canonical SLs. The F-box D3 (MAX2) and D14 proteins are required for SL perception and signal transduction (dashed arrow), which triggers SL response. The transcriptional repressors SMXLs/D53 further involves in the polyubiquitination of D14 and SMXLs/D53 and in the 26S proteasomal degradation (dashed arrow).

**Figure 2.**
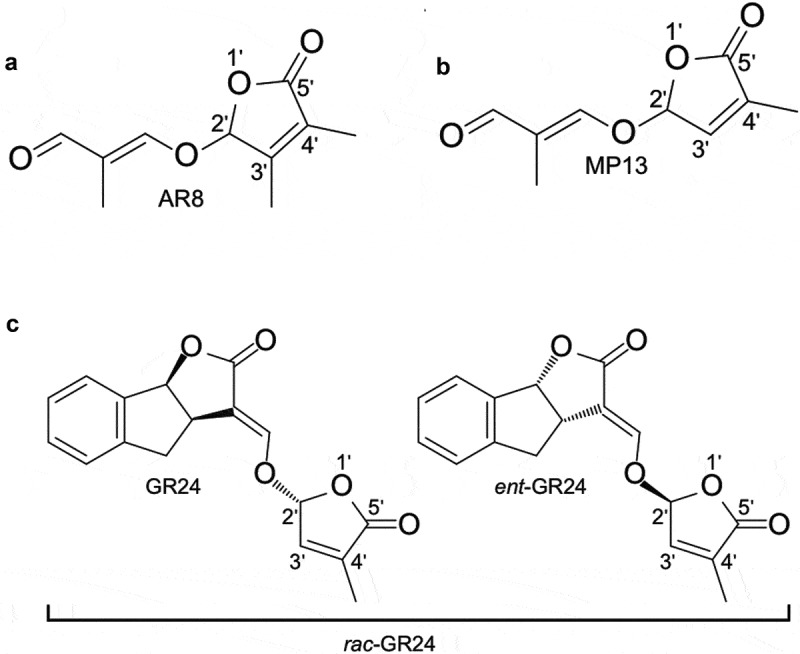
(a) Structure of AR8, a previously described SL analog^22^ containing a methyl group at the C-3ʹ atom of the D-ring. (b) Structure of the SL analog MP13 that differs from AR8 by the absence of C-3ʹ methylation^23^ (c) Structure of the common SL analog *rac*-GR24 consisting of two enantiomers (as shown).

For this purpose, rice Nipponbare (wild type) and *hl-2* (*high-tillering dwarf1-like* (*hl*)) seedlings were grown hydroponically under normal Hoagland’s nutrient solution for 2 weeks with 12-h photoperiod (200 μM m^−2^ s^−1^). Then, rice seedlings were subjected to phosphorus deficiency for another 1 week and treated with 2.5 µM of each SL analog for 6 hours. Total RNA was extracted from rice roots using TRI-Reagent with Direct-zol RNA MiniPrep Kit (Zymo Research). Reverse transcription reaction was performed with the Bio-Rad iScript cDNA Synthesis Kit using 1 µg of total extracted RNA following the manufacturer’s instructions. Primers used for quantitative real-time PCR (qRT-PCR) analysis are listed in (Supplementary Table S1). qRT-PCR was performed in a StepOne™ Real-Time PCR Systems (Applied Biosystems) using SYBR Green Supermix to monitor double-stranded DNA (dsDNA) synthesis following the manufacturer’s instructions. Relative expression levels of genes were determined using a comparative C_t_ method as previously described^24^ and rice *Ubiquitin* (*ubi*) gene (Supplementary Table S1) was used as the internal control to normalize target gene expression.

The results obtained reveal that transcript levels of SL biosynthesis genes *D27, D17* and *D10* were significantly suppressed, and expression of an SL signaling repressor gene *D53* was strongly induced by MP13 and GR24 treatment in both wild type and *hl-2* (*ccd7*) mutant, as compared with mock and methylated AR8 (Figure 3). Indeed, the effect of MP13 and GR24 treatment on *D27, D17, D10*, and *D53* transcript levels was much more pronounced than that of AR8 in both WT and *ccd7* mutant (Figure 3). These results are consistent with the previous findings showing that the expression of SL biosynthesis genes *D27, D17*/*CCD7* and *D10*/*CCD8* is down-regulated by synthetic GR24 treatment and confirming a negative feed-back regulation of SL biosynthetic genes,^8,9,20,25^ while the transcript level of the SL repressor *D53* gene is up-regulated by GR24 treatment.^18^ Moreover, our results indicate that the presence of a methyl group at C-3ʹ in D-ring of SL analog (AR8) reduces the capability to regulate the expression of SL-dependent genes, which is in line with our recent study that shows that this modification leads to a significant decrease in different biological activities.^22^

**Figure 3.**
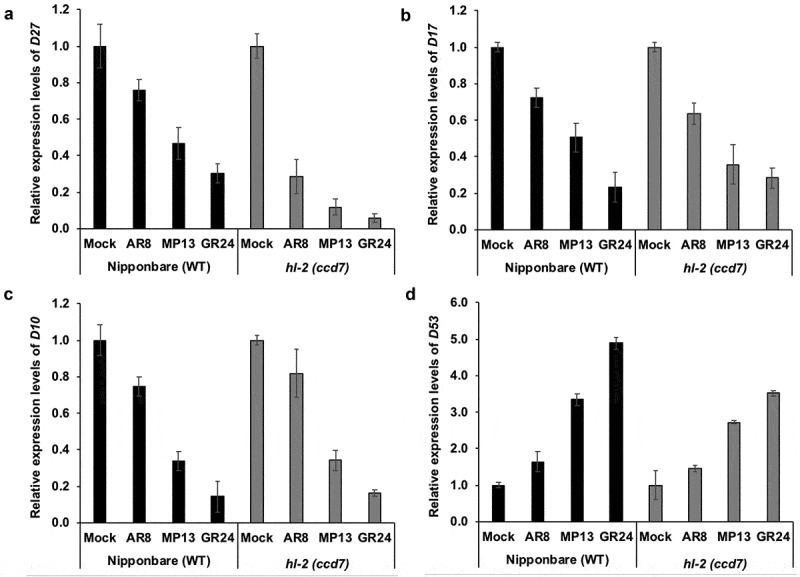
Effect of the treatment with AR8, MP13 and GR24 on transcript levels of the SL biosynthesis genes *D27, D17, D10* (a–c) and the transcriptional repressor *D53* (d). Quantitative real-time PCR measurement was performed with total RNA isolated from roots of Nipponbare (WT) and *hl-2 (ccd7* mutant) rice seedlings grown under phosphorus-deficient conditions. *Ubiquitin* was used as the reference gene, and the transcript levels in the untreated control (mock) were normalized to 1. Bars represent means ± SD (*n*= 3).

Considering the important role of SLs determining plant architecture and interaction with beneficial and detrimental organisms,^13,26^ elucidating the impact of structural diversity on gene expression regulating activity of SLs could be very helpful in developing analogs that can be employed to optimize some important crop traits and resilience to biotic and abiotic stresses. In addition, such studies are expected to help in understanding why plants produce and release structurally diverse SLs.
