# Supplementary material for: Effect of D-ring C-3’ methylation of strigolactone analogs on their transcription regulating activity in rice
Source: Plant Signal Behav. 2019 Sep 25;14(11):1668234. doi: 10.1080/15592324.2019.1668234 (PMC6804695; doi:10.1080/15592324.2019.1668234)
Supplement: Supplemental Material [file kpsb-14-11-1668234-s001.pdf]

Table S1 qRT-PCR primers sequences used in this study

Primer name	Sequences
D27-F	5'-TCTGGGCTAAAGAATGAAAAGGA-3'
D27-R	5'-AGAGCTTGGGTCACAATCTCG-3'
D17-F	5’-TCCACAGGATGTTTGGTTACA-3’
D17-R	5’-GTAGCTTGGGTTTATCGCCG-3’
D10-F	5’-CGTGGCGATATCGATGGT-3’
D10-R	5’-CGACCTCCTCGAACGTCTT-3’
D53-F	5'-CCAAGCAGTTTGAAGCGAC-3'
D53-R	5'-CCGCAAGTTTATCAAAGTCAA-3'
Ubi-F	5'-GCCCAAGAAGAAGATCAAGAAC-3'
Ubi-R	5'-AGATAACAACGGAAGCATAAAAGTC-3'
