# Supplementary material for: Effect of D-ring C-3’ methylation of strigolactone analogs on their transcription regulating activity in rice
Source: Plant Signal Behav. 2019 Sep 25;14(11):1668234. doi: 10.1080/15592324.2019.1668234 (PMC6804695; doi:10.1080/15592324.2019.1668234)
Supplement: Supplemental Material [file kpsb-14-11-1668234-s002.docx]

**Table S1.** List of qRT-PCR primers used in this study
